# Efficient multi-lineage cardiovascular differentiation of human pluripotent stem cells in animal serum-free conditions

**DOI:** 10.1038/s41598-026-56846-2

**Published:** 2026-06-16

**Authors:** Nguyen T. N. Vo, Kelvin Chung, Aishah Nasir, Davor Pavlovic, Chris Denning

**Affiliations:** 1 Translational Medical Sciences, Biodiscovery Institute, University Park, University of Nottingham, Nottingham, NG7 2RD UK; 2 Department of Cardiovascular Sciences, College of Medicine and Health, University of Birmingham, Birmingham, B15 2TT UK

**Keywords:** Biological techniques, Biotechnology, Cardiology, Cell biology, Stem cells

## Abstract

Human induced pluripotent stem cell (hiPSC) technologies offer human-relevant cardiac models for biomedical applications. However, workflows for differentiation of cardiac stromal cells and fabrication of engineered heart tissue (EHT) commonly rely on animal serum, contrary to growing policy demands to reduce use of these products. Applying marker analysis via COL-I, DDR2 and GATA4 for cardiac fibroblasts or CD31, CD34 and CD144 for endothelial cells, we tailored Panexin, a defined serum substitute, to support high efficiency differentiation of cardiac stromal lineages to 85% purity without additional purification steps. We evaluated fabrication of EHTs using hiPSC-cardiomyocytes only (monoculture) or further combined with cardiac fibroblasts and endothelial cells (triculture; 70%:15%:15%, respectively). Panexin poorly supported fabrication and contractility of EHTs, a finding unaltered by modulating spontaneous cardiac myofibroblast activation via TGFβ inhibition. In contrast, human serum enabled fabrication of mono- and tri-culture EHTs, wherein constructs made without TGFβ signalling inhibition delivered the strongest contractile forces and exceeded comparator tissues engineered using animal serum. Our data show that iterative evaluation of serum substitutes, human serum, cell combinations and signalling pathway modulators can mitigate use of animal serum for functional EHT generation, aligning with the UK government’s roadmap for alternative methods.

**Supplementary Information:**

The online version contains supplementary material available at https://doi.org/10.1038/s41598-026-56846-2.

## Introduction

Human induced pluripotent stem cells (hiPSCs) provide a renewable source of human cardiac cell types for disease modelling, drug discovery and safety pharmacology, and emerging regenerative applications. Although hiPSC-derived cardiomyocytes (CMs) are widely used, co-culture with cardiac stromal cells such as cardiac fibroblasts (CFs) and endothelial cells (ECs) often improves tissue organisation, contractile performance and maturation, and can better capture disease phenotypes in vitro^1–4^.

Despite the widespread use of animal serum (e.g. fetal bovine serum, FBS; bovine serum albumin, BSA) in cell culture since the 1950s, its inclusion in differentiation protocols raises several concerns^5^. These include ethical issues, physiological relevance and the undefined nature of animal serum. Introduction of non-human growth factors or cytokines may activate unintended pathways, hindering efforts to identify specific factors responsible for biological effects hence impeding mechanistic studies and protocol optimisation^6–9^. Animal serum can introduce contamination risks (e.g., viruses, prions) and immunogenic responses, complicating therapeutic and regulatory compliance^5,7,10^. These limitations obstruct scaling for industrial applications and regulatory approval. Consequently, there is a shift towards non-animal methods (NAMs), illustrated by the UK government’s recent policy document, “Replacing animals in science: A strategy to support the development, validation and uptake of alternative methods”^11^.

To address these limitations, chemically defined substitutions have been developed. While several serum substitutes are available, many are specialized for the maintenance of pluripotency (e.g., KSR, CTS KnockOut SR) or intended for general-purpose use in both suspension and adherent cultures (e.g., Panexin CD, Panexin Basic). Panexin NTA was selected because it is specifically optimized for adherent cell types, making it more compatible with the metabolic and attachment requirements of our monolayer differentiation protocols. In addition to serum-free substitutes, human serum is a biologically relevant alternative that reflects human physiology, although challenges of variability and availability remain. Of note, engineered heart tissue (EHT) platforms typically rely on animal serum during both fabrication and long-term culture to support compaction, tissue remodelling and contractile maturation.

In this study, we tested two complementary strategies to reduce reliance on animal serum across a multi-lineage cardiac modelling workflow. First, we evaluated Panexin for use in differentiating hiPSC-derived CFs and ECs without the need for further purification or enrichment. Second, we examined whether Panexin or human serum could replace animal serum during EHT fabrication from monocultures of hiPSC-CMs (CM-only) and tricultures (tri-lineage) of hiPSC-CMs, CFs and ECs. This included exploring the impact of prolonged TGFβ pathway modulation of spontaneous cardiac myofibroblast activation on EHT function. While serum-free differentiation of individual cardiac lineages has been reported, a systematic evaluation of serum replacement across both stromal differentiation and functional engineered heart tissue fabrication has not been performed. Here, we provide an integrated and practical framework for replacing animal serum use across a multi-lineage, functional human cardiac tissue workflow, ending with a summary infographic of idealised guidelines.

## Results

### Panexin enables serum-free differentiation of cardiac fibroblasts at high purity

**Fig. 1:**
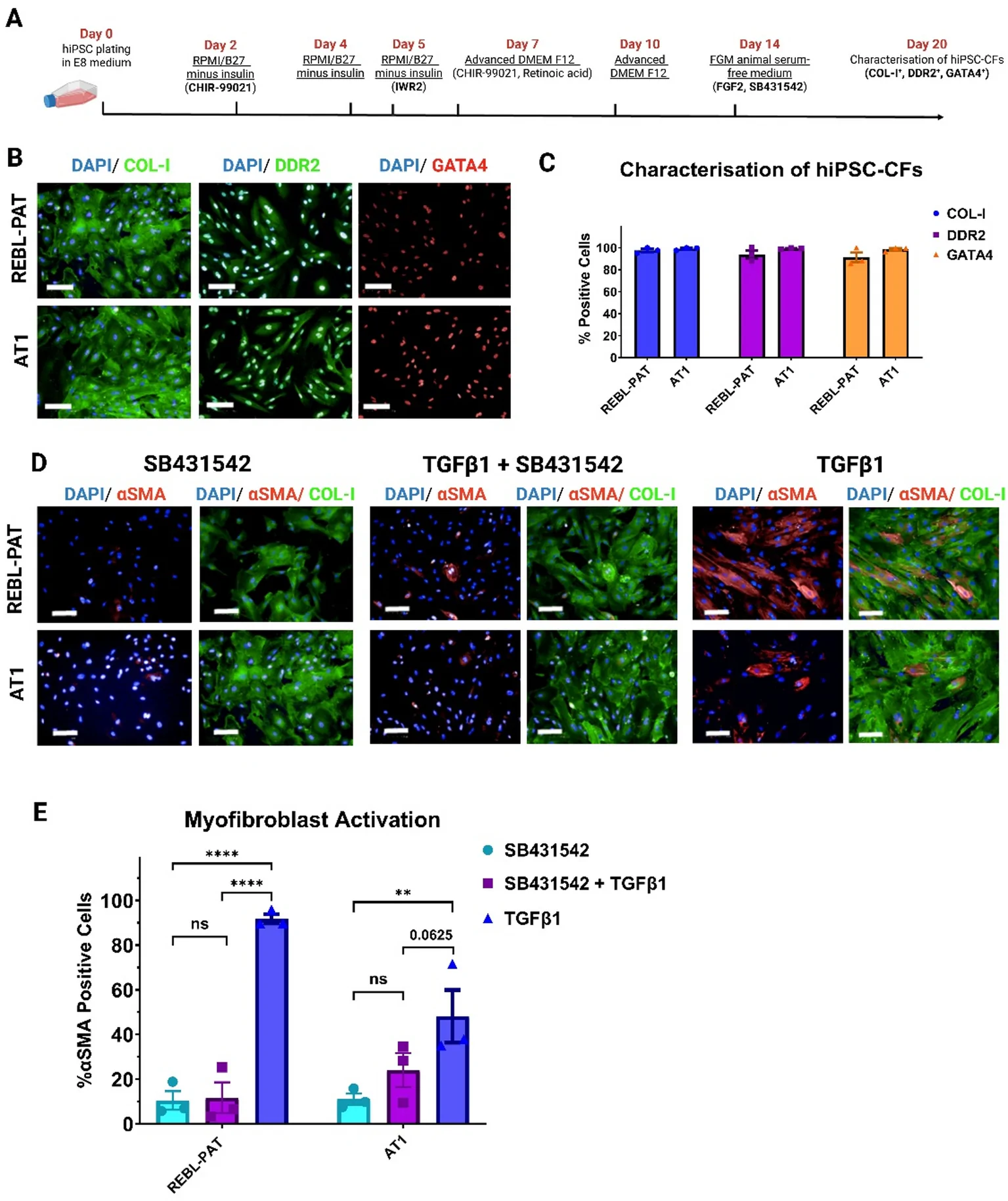
Serum-free differentiation of hiPSC-derived cardiac fibroblasts using Panexin. **(A)** Schematic of the modified protocol. **(B)** Representative immunostaining illustrating the expression of CF-specific markers, including COL-I & DDR2 (green) and GATA4 (red), scale bar: 100 μm. **(C)** Quantification of CF purity presented as mean ± SEM, *N* = 3 biological replicates. **(D)** Myofibroblast activation assay under TGF-β stimulation with and without SB431542. Cells maintained with SB431542 alone were used as the baseline condition. Nuclei (DAPI in blue), COL-I (green), α-SMA (red), scale bar = 100 μm. **(E)** Quantification of αSMA-positive cells presented as mean ± SEM, *N* = 3 biological replicates. Two-way ANOVA with Tukey’s multiple tests; ns, not significant, *p < 0.05, **p < 0.01, ***p < 0.001, ****p < 0.0001. Created in BioRender. Vo, N. (2026) https://BioRender.com/ma2votl.

We had previously optimised cardiomyocyte differentiation of two in-house hiPSC lines, REBL-PAT and AT1^12^, providing the grounding to develop approaches to produce cardiac stromal cell from the same lines. For CFs, we adapted a differentiation protocol from Zhang et al.^13^. Fetal calf serum (FCS) in the Promocell fibroblast growth medium (FGM) was replaced by Panexin at an equivalent volume, generating a serum-free version of FGM (Fig. 1.A). We then trialled differentiation using seeding densities in REBL-PAT from 20,000 to 30,000 cell per cm^2^ (supplementary figure S1) and subsequently applied the working density to the AT1 line. Timelines for differentiation were adapted, with cells replated at day 14 post-differentiation for onward culture in serum-free FGM supplemented with 10 ng/ml FGF2 and 10 µM SB431542.

Endpoint immunostaining at day 20 showed high proportions of cells positive for COL-I, DDR2, and GATA4 in parallel assays, consistent with a cardiac fibroblast-like phenotype (Fig. 1.B). Across biological replicates, more than 90% of cells were marker-positive in both lines (Fig. 1.C), comparable to efficiencies obtained using the serum-based protocol (supplementary figure S1). Taken together, these data show that modified differentiation conditions using Panexin supports high efficiency serum-free production of CFs, which display robust expression of both fibroblast and cardiac-associated markers.

### TGFβ-driven myofibroblast activation is preserved under serum-free conditions and suppressed by SB431542

To assess functional responsiveness, hiPSC-CFs generated under serum-free conditions were treated with TGFβ1 in the presence or absence of the TGFβ receptor inhibitor, SB431542. TGFβ1 induced a pronounced increase in αSMA expression, while co-treatment with SB431542 reduced this induction relative to TGFβ1 treatment alone. Cells maintained with SB431542 alone were used as the baseline rather than a vehicle control. αSMA-positive cell percentage was used as the quantitative readout of myofibroblast activation. Co-treatment with SB431542 markedly reduced αSMA induction, consistent with inhibition of TGFβ signalling (Fig. 1.D).

In the REBL-PAT line, TGFβ1 stimulation resulted in 91.7% αSMA-positive cells. In contrast, cells treated with SB431542 alone or with combined TGFβ1 and SB431542 showed low αSMA expression (< 20%; Fig. 1.E). A similar response was observed in the AT1 line, with 45.2% αSMA-positive cells following TGFβ1 treatment compared with 11% for SB431542 alone and 22.7% for combined TGFβ1 and SB431542. Overall, hiPSC-CFs generated under Panexin-based serum-free conditions retain expected responsiveness to TGFβ signalling. The modulation of myofibroblast activation closely parallels that observed in serum-based differentiation protocols (supplementary figure S1), supporting the functional equivalence of CFs derived using Panexin.

### Panexin supports serum-free endothelial differentiation without purification

**Fig. 2:**
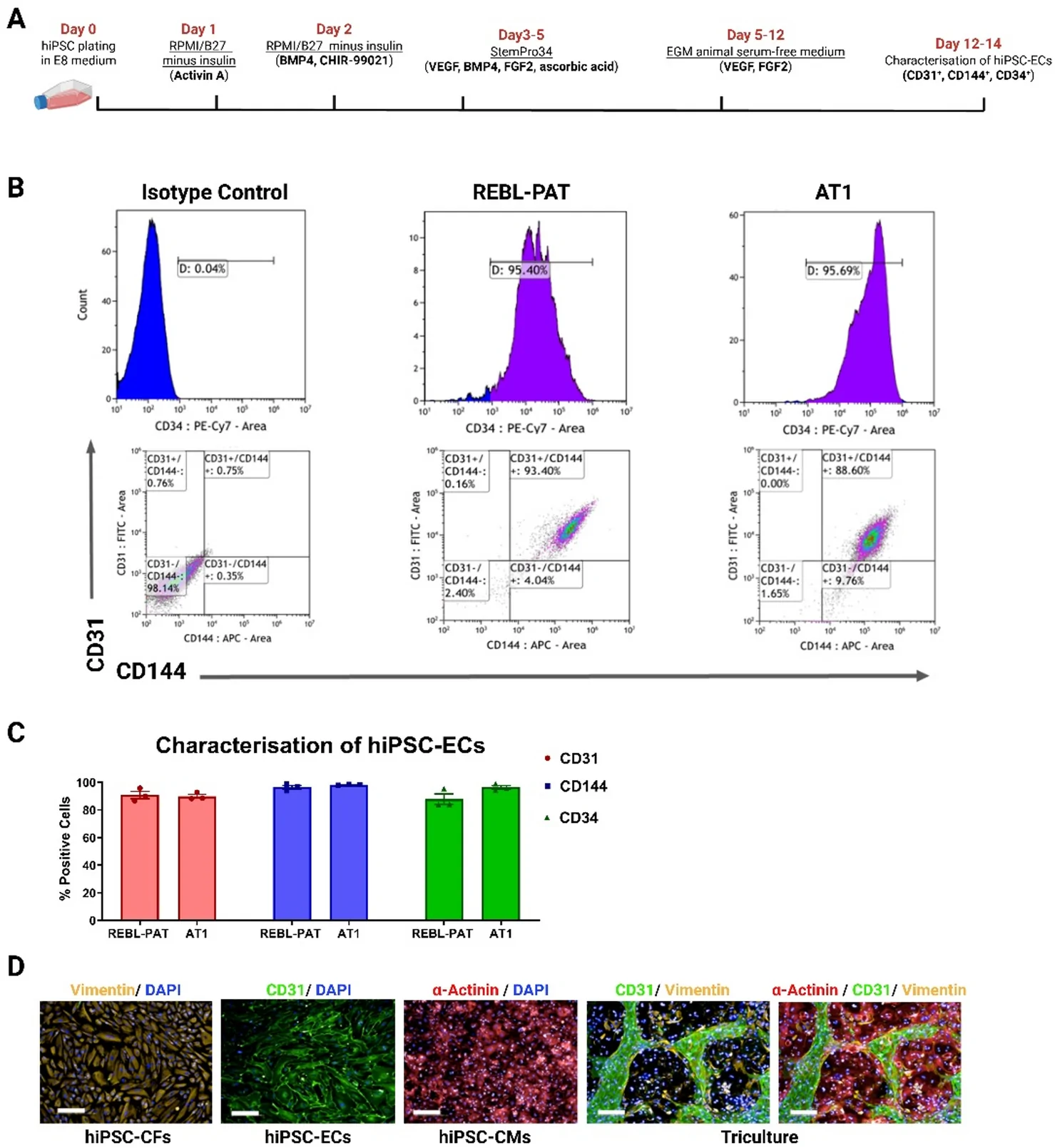
Serum-free differentiation of hiPSC-derived endothelial cells using Panexin. **(A)** Schematic of the modified protocol. **(B)** Representative flow cytometry for EC-specific markers CD31, CD144 (VE-Cadherin) and CD34 in REBL-PAT and AT1. **(C)** Quantification of EC purity generated by the serum-free protocol, presented as mean ± SEM, *N* = 3 biological replicates. Two-way ANOVA with Tukey’s multiple tests; ns, not significant. **(D)** Representative images illustrating endothelial network-like organisation in a triculture of REBL-PAT-derived hiPSC-ECs, hiPSC-CFs and hiPSC-CMs, Vimentin (yellow), CD31 (green) and α-actinin (red), scale bar 200 μm. Created in BioRender. Vo, N. (2026) https://BioRender.com/ma2votl.

Endothelial differentiation was adapted from our previously published protocol^14^ by replacing bovine brain extract (BBE) and fetal bovine serum (FBS) in the commercial Lonza EGM™ Endothelial Cell Growth Medium BulletKit™ with an equivalent volume of Panexin to generate a serum-free formulation of EGM (Fig. 2.A). Optimisation of seeding density was systematically evaluated in the REBL-PAT hiPSC line, with densities ranging from 20,000 to 40,000 cells per cm². Flow cytometry analysis demonstrated that seeding density influenced endothelial differentiation efficiency under both Panexin (serum-free) and serum-based conditions, and that optimised densities were required to achieve > 85% marker-positive cells (Supplementary Fig. S2). The optimised seeding parameters identified in REBL-PAT were subsequently applied to the AT1 line for endothelial differentiation experiments.

Following replating and maturation, flow cytometry on day 13 showed greater than 85% positive cells for CD31, CD144 and CD34 obtained in both REBL-PAT and AT1 without the need for magnetic- or FACS-based purification (Fig. 2.B - C). Preserved endothelial functionality was illustrated in triculture monolayers of CMs, CFs and ECs, wherein vascular-like networks were evident after 7 days of co-culturing (Fig. 2.D).

### Panexin does not support functional contractile monoculture hiPSC-CM EHTs

Strip-format fibrin-based EHTs^15^ are widely used to assess contractile responses of human and animal cardiac tissues in 3D format. As this protocol requires FCS (10% v/v) during fabrication and horse serum (10% v/v) during maintenance culture, we evaluated whether Panexin could replace animal serum during fibrin-based EHT formation and long-term culture (supplementary figure S3.A & B).

**Fig. 3:**
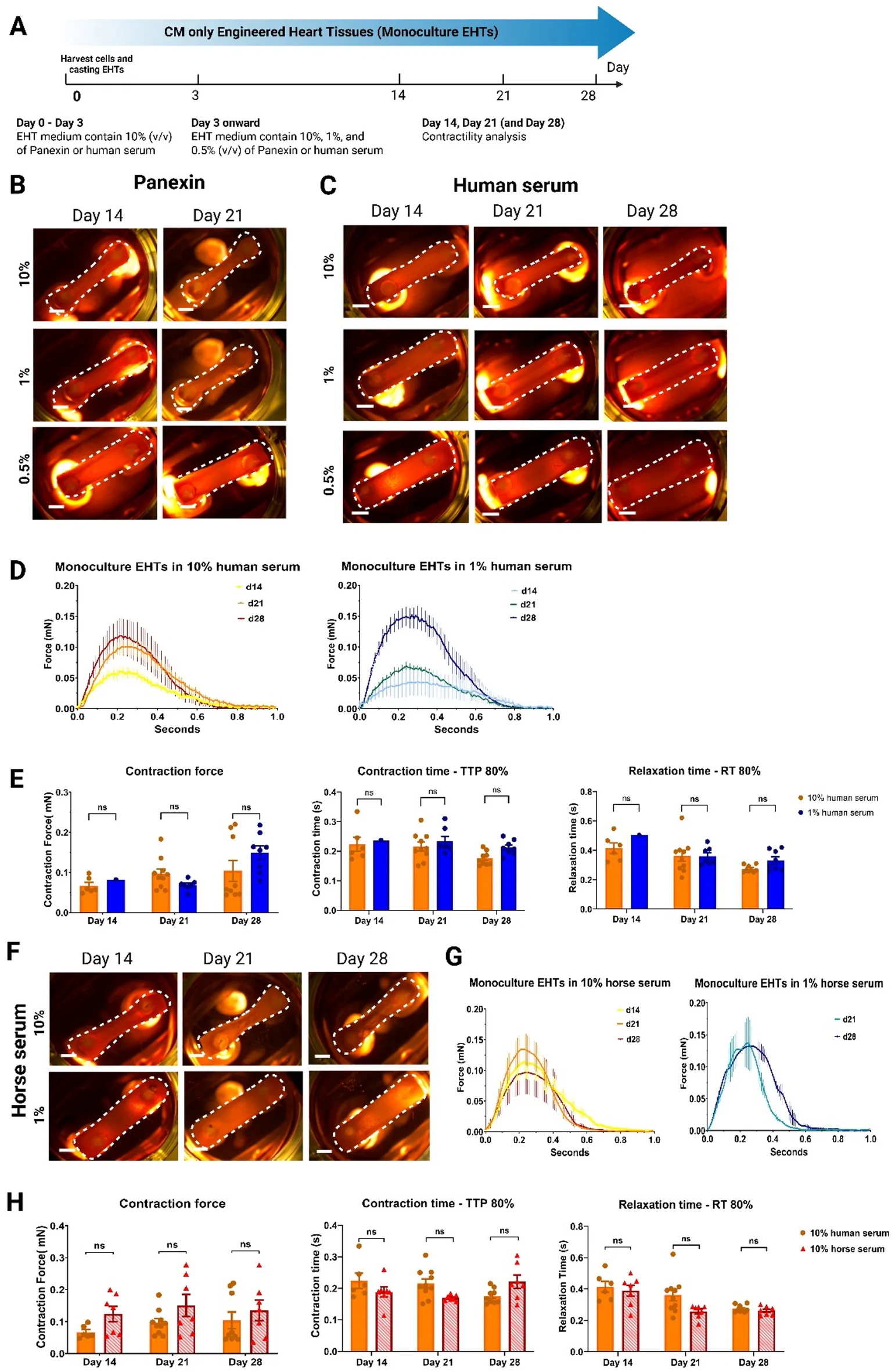
Serum replacement in monoculture EHTs from cardiomyocyte only. **(A)** Workflow schematic. Representative morphology of monoculture EHTs over time in Panexin conditions **(B)**, and human serum conditions **(C)**, scale bar 2 mm. **(D)** Contraction traces of monoculture EHTs in 10% and 1% v/v human serum. **(E)** Comparison of contraction force, contraction time, and relaxation time between low (1% v/v) and high (10% v/v) human serum concentrations. **(F)** Representative morphology in monoculture EHTs in horse serum, animal serum control. **(G)** Contraction traces of monoculture EHTs cultured with horse serum at high (10% v/v) and low (1% v/v) concentrations. **(H)** Comparison of contraction force, contraction time, and relaxation time between 10% (v/v) human serum and 10% (v/v) horse serum. Data in panels **E** and **H** are presented as mean ± SEM. Datapoints are technical EHT replicates to show experimental variation, but statistical comparisons were performed using biological replicates (*N* = 3). Two-way ANOVA with Tukey’s multiple-comparisons test; ns, not significant; *p < 0.05, **p < 0.01. Created in BioRender. Vo, N. (2026) https://BioRender.com/ma2votl.

All EHT experiments were performed using the AT1 hiPSC line. Cardiomyocytes were differentiated from AT1 cells and used to generate CM-only EHTs, also termed monoculture EHTs, and maintained in medium supplemented with Panexin at 10%, 1%, or 0.5% (v/v) (Fig. 3A). Tissue morphology and contractile activity of monoculture EHTs in Panexin-supplement medium were monitored up to 21-day post-fabrication, a time window during which functional EHTs typically exhibit stable and reproducible contractile activity, using serial imaging and video-based force analysis (supplementary figure S3.C-D).

Morphologically, EHTs cultured with 10% Panexin exhibited defined borders and progressive remodelling from an initial rectangular geometry to a characteristic dumbbell shape (Fig. 3.B), similar to tissues generated using animal serum (Fig. 3.F). Despite preserved structural integrity, these EHTs failed to exhibit spontaneous beating or contractile responses upon electrical pacing at 1 Hz. Reducing Panexin from 10% to 1% or 0.5% (v/v) compromised EHT structural integrity, remodelling, and function, with no detectable contractile activity, either in spontaneous or paced conditions. Tissue formation and stability were further reduced under serum-free (0%) conditions, where low levels of cell attachment to the silicon posts were observed, and remaining tissues frequently detached or failed to exhibit contractile responses during maturation (supplementary figure S3.D). Together, these findings indicate that, while Panexin supports morphological remodelling of CM-only EHTs at higher concentrations, it is insufficient to sustain functional contractility under all tested concentrations.

### Human serum supports functional EHT fabrication and contractility

Given the limitations of Panexin in supporting functional monoculture EHTs, using AT1-derived CMs, we next investigated whether human serum could replace animal serum under otherwise identical EHT fabrication and culture conditions. In contrast to Panexin-supported tissues with absent functional contractility, human serum-supported EHTs demonstrated stable structural integrity and functional activity, enabling stable and consistent contractile activity by day 14, which permitted extended monitoring up to 28 days post-fabrication. EHTs generated in 10% or 1% human serum displayed well-defined borders and robust remodeling, whereas tissues formed in 0.5% human serum showed partial structural remodeling but failed to support stable functional contractility (Fig. 3.C).

Functional assessment revealed that monoculture EHTs cultured with 1% or 10% human serum exhibited stable spontaneous contractions by day 14, with contractile parameters stabilising by approximately day 21 (Fig. 3D, E). This performance was broadly comparable to tissues produced using animal sera (Fig. 3F, G), though the onset of detectable contractile activity in the 1% horse serum condition did not occur until day 21. At the higher 10% v/v concentration, contractile parameters remained comparable between human and animal serum sources (Fig. 3H). These results demonstrate that human serum can effectively support both morphological maturation and functional contractility of CM-only EHTs, providing a human-relevant alternative to animal serum.

### Omission of TGFβ inhibition enhances triculture EHTs contractility

Cardiac fibroblasts and endothelial cells can confer a positive impact on cardiomyocyte function and maturation through extracellular matrix deposition and paracrine signalling^1–3^. We therefore investigated whether incorporation of cardiac stromal cells would influence morphological, structural, or functional properties of EHTs under animal serum-free conditions. Triculture EHTs were generated using the AT1 line with a composition of 70% CMs, 15% CFs, and 15% ECs, a ratio previously optimized for the structural and functional maturation of in vitro human heart tissues^3^.

To support all three cell types within a single construct, a universal EHT culture medium was used for both mono- and tri-culture tissues throughout this study. This medium consisted of DMEM supplemented with 1% penicillin/streptomycin, 0.1% insulin, 0.1% aprotinin, and either Panexin or serum (human or horse) at 10% or 1% (v/v), with the remaining volume made up by DMEM. This formulation was selected to maintain compatibility across CMs, CFs, and ECs while enabling direct comparison of serum and serum-replacement conditions.

**Fig. 4:**
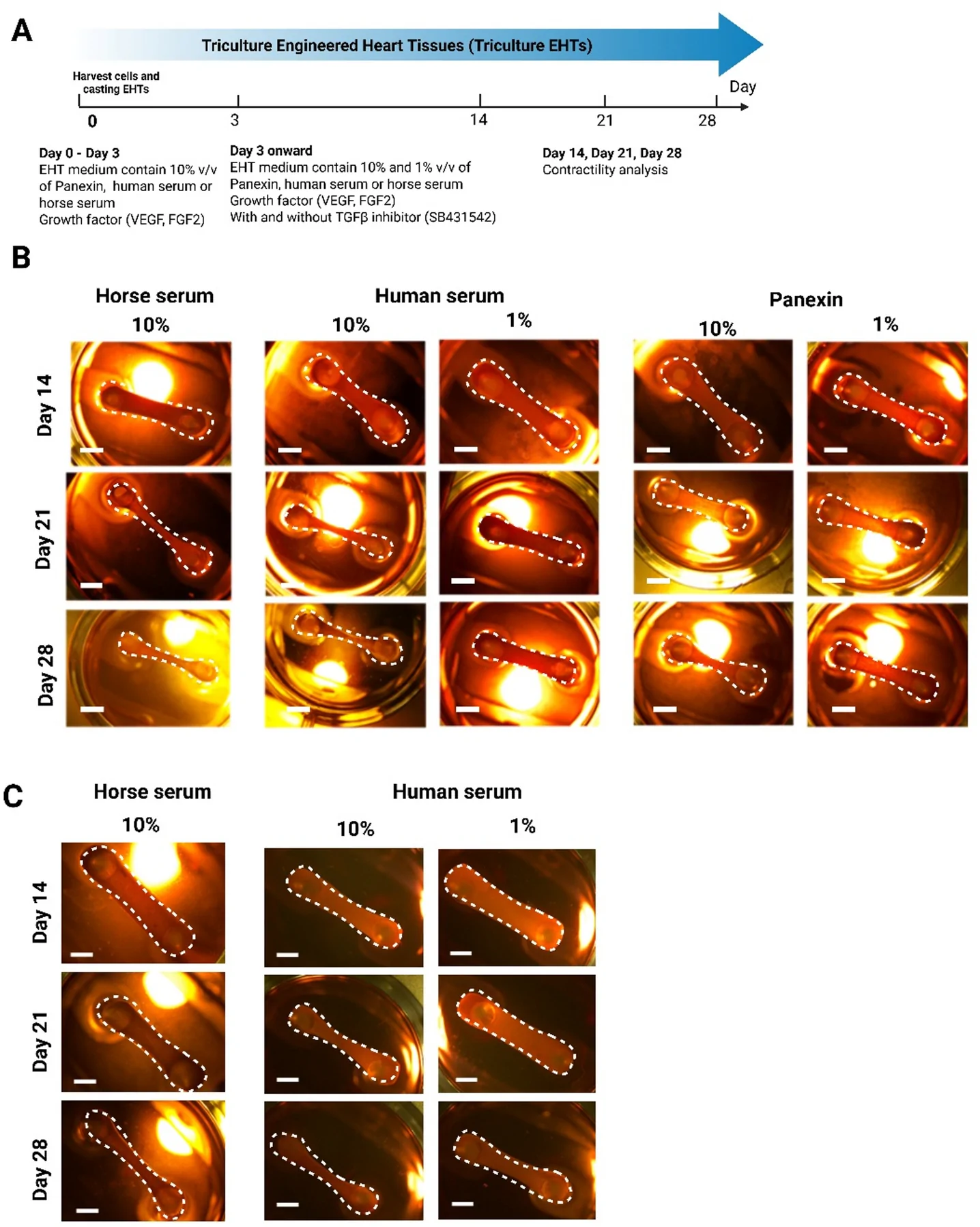
Triculture EHT morphology under serum replacement. **(A)** Workflow schematic for triculture EHTs. Representative morphology of EHTs over time across serum and serum-replacement conditions with SB431542 **(B)** and without SB431542 **(C)**, scale bar 2 mm. Created in BioRender. Vo, N. (2026) https://BioRender.com/ma2votl.

Based on poor performance in previous experiments, Panexin and human serum concentrations of 0% and 0.5% were excluded, as was 1% horse serum. Vascular endothelial growth factor (VEGF; 30 ng/mL) and fibroblast growth factor 2 (FGF2; 10 ng/mL) were added into the universal EHT medium culture to support ECs and CFs within the triculture constructs. To modulate spontaneous myofibroblast activation, EHTs were cultured in the presence or absence of the TGFβ receptor inhibitor SB431542 (10 µM) (Fig. 4.A; supplementary figure S4).

**Fig. 5:**
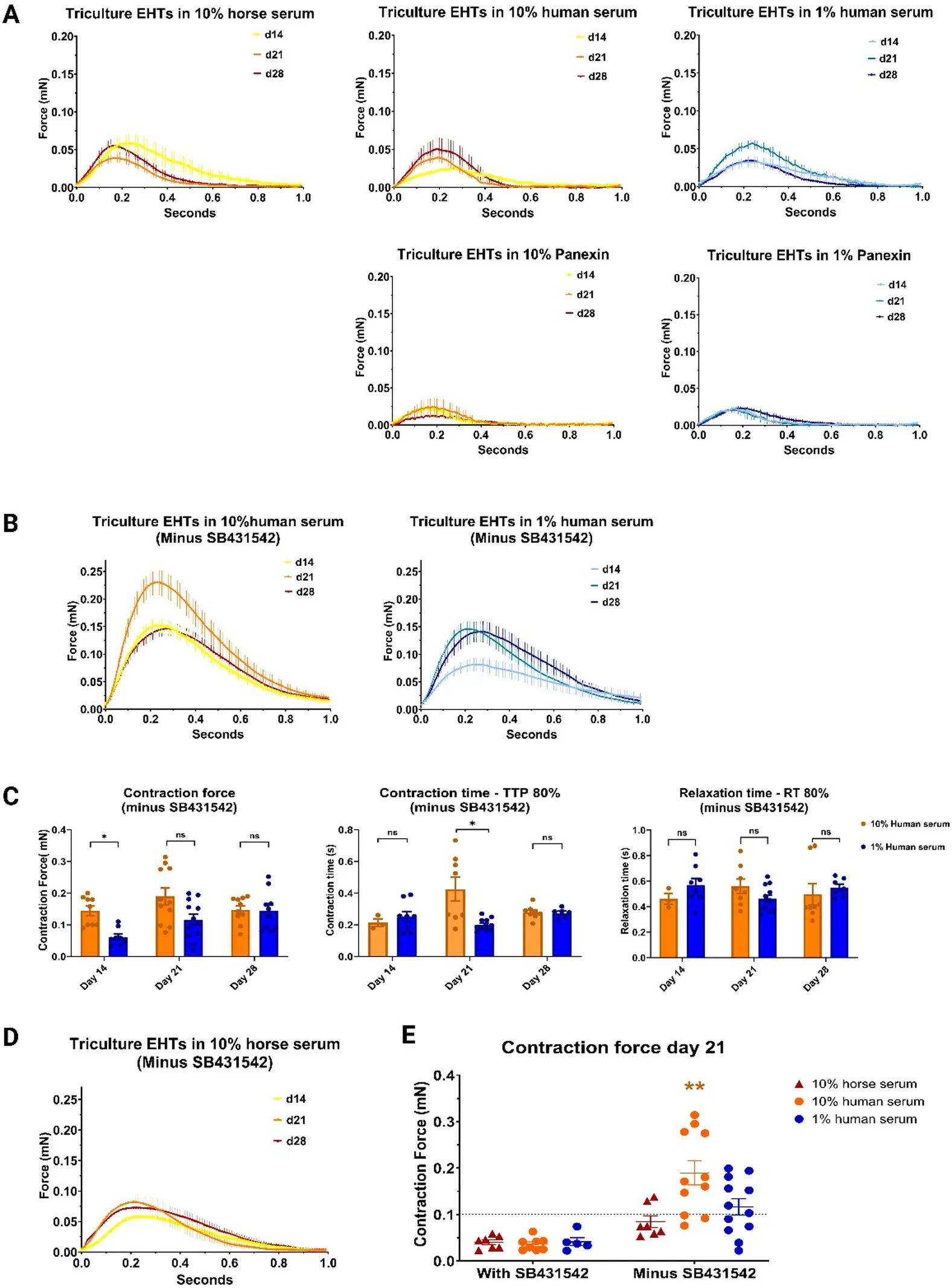
Contractile analysis of triculture EHTs and effect of SB431542. **(A)** Mean contraction traces of triculture EHTs across serum and serum-replacement conditions in the presence of SB431542. **(B)** Mean contraction traces of triculture EHTs cultured with 10% and 1% (v/v) human serum in the absence of SB431542. **(C)** Comparison of contraction force, contraction time, and relaxation time of triculture EHTs cultured with 10% and 1% (v/v) human serum in the absence of SB431542. Two-way ANOVA with Tukey’s multiple-comparisons test; ns, not significant; *p < 0.05. **(D)** Mean contraction traces of triculture EHTs cultured with 10% horse serum in the absence of SB431542. **(E)** Direct comparison of contraction force at day 21 post-fabrication across all serum conditions in the presence and absence of SB431542. Two-way ANOVA with Sidak’s multiple-comparisons test; colour-coded according to serum condition; ns, not significant; *p < 0.05, **p < 0.01. In C and E, data are presented as mean ± SEM. Datapoints are technical EHT replicates to show experimental variation, but statistical comparisons were performed using biological replicates (*N* = 3). Created in BioRender. Vo, N. (2026) https://BioRender.com/ma2votl.

Across all tested conditions (1% or 10% serum or Panexin, with or without SB431542), triculture EHTs exhibited well-defined boundaries and pronounced remodelling by day 14. This further increased by day 21 (Fig. 4.B-C), indicating a positive structural contribution from the cardiac stromal populations. This observation is consistent with previous reports that cardiac stromal cells can support structural organisation and maturation in multicellular cardiac tissues^1,3^. However, marked differences in contractile performance were observed depending on SB431542 treatment (Fig. 5).

In the presence of SB431542, contraction forces were substantially reduced across all conditions, reaching only 0.02 mN in Panexin-supported EHTs, and around 0.05 mN in human serum-supported and horse serum-supported EHTs (Fig. 5.A). In contrast, omission of SB431542 restored contractile performance in both 1% and 10% human serum conditions, with forces attaining or exceeding 0.1–0.15 mN by day 21 (Fig. 5.B-C). These values were comparable to, or greater than, those observed in monoculture EHTs (Fig. 4).

Omission of SB431542 in triculture EHTs cultured with horse serum also showed an increase in contractile force (Fig. 5.D). Direct comparison demonstrated that triculture EHTs cultured with 1% human serum closely aligned with the contractile performance observed in the 10% v/v animal serum or human serum conditions from day 21 onward (Fig. 5.E). This comparative analysis across all serum conditions revealed a pronounced negative impact of SB431542 on contraction force. Despite the positive contribution of cardiac stromal cells, triculture EHTs cultured with Panexin did not show meaningful recovery of contractile force (~ 0.04 mN) following SB431542 removal in preliminary validation experiments (Supplementary Fig. S5), which remained insufficient for functional studies requiring robust contractile output.

## Discussion

**Fig. 6:**
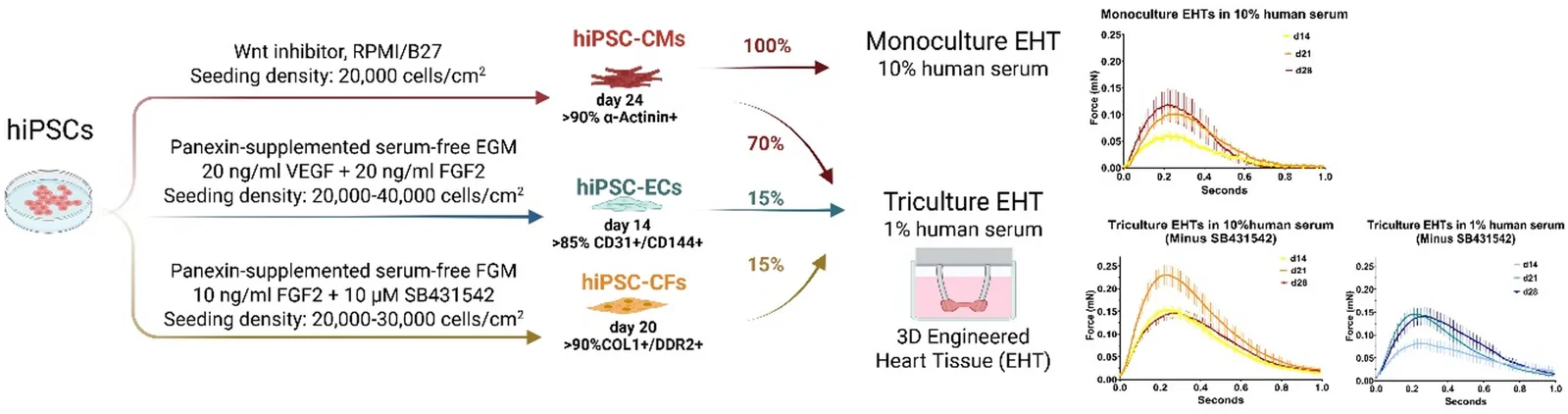
Summary of serum-free stromal differentiation and optimised serum conditions for engineered heart tissue assembly. Schematic overview of the workflow developed in this study. Human iPSCs were differentiated into cardiomyocytes (CMs), endothelial cells (ECs), and cardiac fibroblasts (CFs) using serum-free protocols, wherein fetal bovine serum was replaced by Panexin for stromal lineages. For engineered heart tissue (EHT) fabrication, 10% v/v human serum efficiently supported morphological remodelling and functional contractility in CM-only EHTs. Triculture EHTs assembled from CMs (70%), ECs (15%), and CFs (15%) showed enhanced structural organisation and contractile performance under low human serum conditions (1% v/v) in the absence of prolonged TGFβ receptor inhibition. Representative contraction traces illustrate functional differences between monoculture and triculture EHTs under optimised conditions. Created in BioRender. Vo, N. (2026) https://BioRender.com/ma2votl.

As summarised in Fig. 6, this study provides a guide on the conditions required to negate use of animal serum in differentiating cardiovascular lineages from hiPSC and then fabricating these into mono- or tri-culture engineered heart tissues.

Panexin NTA is defined substitute without growth factors, hydrolysates or peptones and is reported to be free of animal-derived components, making it an attractive candidate for animal-free cell culture systems. These advances in serum-free differentiation protocols for cardiac stromal cells build on our previous work^14^ by demonstrating scalable, purification-free generation of both CFs and ECs using Panexin. Compared to previously reported serum-free cardiovascular differentiation protocols, the present method offers distinct advantages in simplicity and scalability. Our approach achieves high-efficiency stromal differentiation (~ 85% purity) using similar marker profiles to established protocols^3,13,16,17^, but notably removes the requirement for FACS-based purification often needed to ensure endothelial cell purity. Furthermore, our hiPSC-ECs demonstrate functional network-like organization, while hiPSC-CFs maintain a non-activated phenotype similar to those described by Zhang et al.^13^. While functional performance in 3D EHTs remains limited under growth-factor-free conditions, this study provides a streamlined foundation for the sorting-free generation of multi-lineage cardiac cells. In addition, although CF and EC identity was supported by marker expression, TGFβ-responsive αSMA induction and endothelial network-like organisation in co-culture, future studies should include dedicated functional assays, such as CF scratch/wound-healing assays and quantified EC tube-formation or network-formation assays, to further validate stromal cell function.

While hiPSC-CFs and hiPSC-ECs differentiated with Panexin demonstrated high marker positivity, we acknowledge the absence of temporal profiling as a limitation, given that certain markers may be shared with upstream progenitors. Nevertheless, our endpoint marker data, coupled with the use of lineage-specific induction media and functional validation, support the assignment of committed phenotypes. Future ‘omics’ studies will be essential to definitively map the differentiation kinetics and commitment windows in these serum-free conditions.

Although Panexin supported robust differentiation of CFs and ECs, it did not support functional contractile maturation in fibrin-based CM-only EHTs, indicating that this defined serum substitute may lack key components required for three-dimensional tissue assembly and electromechanical coupling. In established fibrin-based EHT systems, reproducible contractile activity is typically evident by approximately 14 days post-casting and stabilises by around 21 days^15,18–20^. In the present study, Panexin-supported monoculture EHTs failed to exhibit spontaneous or paced contractions within this recognised functional maturation window. Given the absence of measurable contractile activity by day 21, further extension of culture duration under Panexin conditions was not pursued. Panexin NTA Pharma Grade is a defined serum substitute for adherent cells containing purified proteins, lipids, salts, amino acids, trace elements, attachment factors and hormones, but no added growth factors, hydrolysates or peptones. Therefore, while suitable for controlled 2D stromal differentiation, Panexin may lack sufficient soluble factors or matrix-remodelling cues required for fibrin-based EHT compaction, ECM maturation and electromechanical coupling. Although incorporation of cardiac stromal cells improved structural remodelling in triculture EHTs, contractile performance in Panexin-based conditions remained inconsistent and insufficient compared with animal serum controls^15,20^. Previous studies indicate that serum-free 3D cardiac tissue generation requires optimisation of media, matrix and maturation cues, including growth-factor supplementation, thyroid hormone and electrical conditioning^21,22^. This interpretation was supported by our no-serum EHT observations, where low attachment to the silicon posts and tissue detachment were observed during culture. Future transcriptomic, proteomic and ECM-focused analyses will be required to identify the serum-derived factors or altered pathways contributing to the limited force generation observed in Panexin-supported EHTs. These findings suggest that the transition to defined xeno-free EHT models requires more optimizations of the fabrication protocol and environmental cues, rather than the direct substitution of animal serum components into existing workflows.

Human serum is a functional alternative for EHT fabrication and maintenance. At low concentration (1%), human serum consistently supported EHT formation and contractile force comparable to animal-serum controls, while potentially reducing non-physiological high-serum effects. Notably, low concentrations of human serum may minimise hypertrophic and fibrotic responses compared to higher serum concentrations, reducing experimental artefacts and enhancing applicability for disease using hiPSCs models. This study showcases the utility of human serum in supporting cardiac tissue engineering while maintaining physiological relevance and also align with recent serum-free studies^17,23^. The use of human serum rather than animal-derived alternatives provides a human-relevant, xeno-free environment that preserves the complex biochemical cues, such as IGF-1, TGFβ, and lipids, necessary for functional maturation and clinical translatability^21,24^. Future studies should examine maturation markers and omics-based profiles to determine whether human serum provides additional maturation benefits over animal-serum alternatives or defined substitutes.

Although challenges remain to using human serum, such as batch-to-batch variability, limited scalability, and logistical constraints, particularly hormone cycles for female donors^17,25^, the closer physiological relevance and the ability to use low concentrations make it an attractive interim option for NAM-aligned cardiac tissue models. Moreover, human serum is subject to extensive donor screening and quality control procedures, including viral testing (e.g., HIV, hepatitis B and C), sterility, and endotoxin assessment at the blood bank and manufacturing stages^7,26,27^. Such standardised screening and processing steps can mitigate safety risks and support translational use, particularly when combined with low serum concentrations and defined culture conditions.

A key finding with practical implications is the reduction in triculture EHT contractile force following prolonged exposure to the TGFβ receptor inhibitor SB431542. SB431542 is widely used to limit myofibroblast activation and has been shown to protect cardiomyocytes from contractile dysfunction driven by excessive TGFβ signalling under fibrotic or pathological stress conditions^13,17,24^. In contrast, our data indicate that sustained TGFβ pathway inhibition compromises contractile performance under non-fibrotic baseline conditions. A plausible explanation is disruption of essential extracellular matrix (ECM) remodelling, as SB431542 inhibits fibroblast-mediated collagen gel contraction, a process required for tissue compaction and mechanical conditioning^22,28,29^. Importantly, this observation does not contradict reports in which SB431542 improved or preserved contractility in human 3D cardiac models, as those studies typically employed short-term exposure during or after pathological stimulation^30–32^.

In contrast, prolonged exposure (up to 28 days) in our healthy triculture EHTs may reveal context-dependent effects on physiological ECM dynamics. These findings highlight the importance of duration- and context-specific optimisation of TGFβ inhibition in human three-dimensional cardiac constructs^33^. Future work should therefore explore strategies such as timed dosing or reduced inhibitor concentrations to balance suppression of excessive fibrosis with preservation of necessary matrix mechanics. Our finding in using low human serum concentrations (1% v/v) to sufficiently support functional triculture EHTs further underscores the need to interpret TGFβ modulation within defined experimental frameworks. All EHT comparisons were performed using the AT1 line to ensure consistency throughout the longitudinal optimization process. Future studies will focus on extending these 3D findings to additional hiPSC genetic backgrounds.

Overall, our findings support a stepwise transition away from animal serum by: (i) using Panexin for scalable, purification-free cardiac stromal lineage differentiation; (ii) using low-concentrations of human serum for functional EHT generation; and (iii) re-optimising anti-fibrotic interventions such as SB431542 within triculture tissues. Together, these advances align with 3Rs principles and strengthen the translational relevance and reproducibility of hiPSC-based cardiac models. Although significant progress has been made, further research is needed to refine protocols for 3D tissue generation, enhance functional maturation, and address practical challenges associated with serum replacement. These efforts will pave the way for the development of reproducible, ethical, and clinically relevant cardiac models, advancing both basic research and translational applications in regenerative medicine.

## Material & methods

A complete list of reagents, media, supplements, and their suppliers is provided in Supplementary key resource table.

### Monolayer cardiac differentiation

REBL-PAT and AT1 hiPSC lines have been previously established and characterized^12,34^, showing expression of pluripotency markers (OCT4, SOX2, NANOG, SSEA4, TRA-181) and normal karyotypes (AT1 46,XX; REBL-PAT 46,XY). These hiPSC lines were used between passages 27–40 in Essential 8™ medium, with passaging every 2–3 days at 70–80% confluence using TrypLE Select. Cardiac differentiation was performed using a monolayer protocol adapted from reference^12^. Monolayer cardiac differentiation began by seeding hiPSCs on day 0 and transitioning to StemPro-34 with BMP4 and Activin A as specified. WNT modulation was achieved using KY02111 and XAV939, followed by culture in RPMI/B27 (with insulin) from day 7–9 onward. Cardiomyocytes were dissociated at day 12 using TrypLE Express and collected by centrifugation at 300×g for 5 min. Prior to EHT fabrication, hiPSC-cardiomyocytes were characterized for cardiac-specific marker expression to ensure high differentiation efficiency of greater than 90%. Panexin was not investigated during hiPSC-CM differentiation because the established cardiomyocyte differentiation protocol was already serum-free and did not require FBS/FCS supplementation.

### Cardiac fibroblast differentiation

Cardiac fibroblast differentiation was adapted from Zhang et al.^13^ with replacement of FCS by Panexin NTA Pharma Grade (Panexin, PAN Biotech, #P04-95750P). The manufacturer’s datasheet states that Panexin NTA Pharma Grade is a defined serum substitute for adherent cells. It contains purified proteins, lipids, salts, amino acids, trace elements, attachment factors, hormones, and trace human-derived components (< 2% w/v), with no added growth factors, hydrolysates, or peptones (Panexin NTA Pharma Grade, Xeno-Free Defined Serum Substitute for Adher – Ilex Life Sciences). Following WNT activation and inhibition phases, cells were replated and matured in fibroblast growth medium (FGM, PromoCell #C-23130) supplemented with Panexin (replacing fetal calf serum), 10 ng/ml FGF2 and 10 µM SB431542, where indicated. Medium was refreshed every 2 days. The cells were characterised on day 20 post-differentiation using fibroblast markers (COL-I and DDR2) and a cardiac marker (GATA4).

### Endothelial differentiation

Endothelial differentiation was adapted from previously published protocol^14^ with substitution of FBS and BBE by Panexin. Briefly, after mesoderm induction, cells were treated with BMP4, FGF2 and VEGF and replated at day 7 in endothelial growth medium (EGM, Lonza #CC-3124) supplemented with Panexin, plus 20 ng/ml VEGF and 20 ng/ml FGF2. Medium was refreshed every 2 days. The differentiated cells were characterised at day 13 post-differentiation by flow cytometry for endothelial-specific markers, including CD31 (PECAM1), CD34, and CD144 (VE-Cadherin).

### Myofibroblast activation assay

To induce myofibroblast activation, hiPSC-derived cardiac fibroblasts at day 22 post-differentiation were treated with recombinant human TGFβ1 (10 ng/mL) for 48 h. Where indicated, cells were co-treated with the TGFβ receptor inhibitor SB431542 (10 µM) to suppress pathway activation. Cells maintained in SB431542 without TGFβ1 were used as the SB431542-maintained baseline, consistent with the established maintenance strategy used to preserve a non-activated hiPSC-CF phenotype. A separate vehicle-only condition was not included. Therefore, this assay was used to assess TGFβ1-induced αSMA expression and its suppression by SB431542 co-treatment, rather than the independent effect of SB431542 withdrawal alone. Following treatment, cells were processed for immunostaining and quantitative analysis of αSMA expression.

### Fabrication of EHTs

Engineered heart tissues (EHTs) were generated following protocols^15,20^. Briefly, strip-format fibrin-based EHTs were generated in agarose casting moulds with PDMS racks. A master mix containing 1 × 10⁶ cells per tissue (CM-only or triculture at 70:15:15 cm: CFs: ECs) was combined with fibrinogen and thrombin and cast between posts. After polymerisation, tissues were cultured for 3–4 weeks in EHT medium containing either horse serum (control), human serum, or Panexin at indicated concentrations. A single lot of human serum were used across experiments. Human Serum derived from pooled donors and was heat inactivated. VEGF (30 ng/ml) and FGF2 (10 ng/ml) were added for triculture tissues, and SB431542 (10 µM) was included or omitted as indicated. The video-optical recording and contraction analysis were conducted by EHT Technologies and Consulting Team Machine Vision (CTMV) software. Based on established EHT literature, functional contractile maturation is typically evident by 14–21 days post-fabrication^18,19^. Therefore, functional assessments were focused within this time window. Contraction time (TTP 80%) was defined as the time interval between 80% of peak force during the ascending phase and the time of peak force. Relaxation time (RT 80%) was defined as the time interval between peak force and 80% of peak force during the descending phase. Parameters were automatically calculated using CTMV software based on silicone post deflection and force calibration settings. Each biological replicate represents an EHT fabrication performed on different independent differentiation. For EHTs, individual datapoints are shown to illustrate within-batch variability, but statistical comparisons were performed using biological replicates from *N* = 3 independent EHT fabrications.

### Immunocytochemistry

Cells were fixed in 4% paraformaldehyde, permeabilised with 0.05% Triton X-100, blocked with 4% goat serum, and incubated with primary antibodies overnight at 4 °C followed by Alexa Fluor secondary antibodies. Nuclei were stained with DAPI. To ensure unbiased quantification, images were acquired using the Operetta^®^ High-Content Imaging System (PerkinElmer) and processed using Harmony^®^ High-Content Imaging and Analysis Software (PerkinElmer). Automated image analysis workflows were utilized to quantify marker-positive cells across 75–90 random fields per replicate. This high-throughput approach ensured that a minimum of 5,000 cells were analysed per condition (across *N* = 3 independent biological replicates) to determine the percentage of marker-positive cells.

### Flow cytometry

Cells were fixed in 4% paraformaldehyde, permeabilised with 0.05% Triton X-100, blocked with 1% BSA in PBS. The cell pellet was transferred to flow cytometry tubes or V-bottom 96-well plates and then incubated with antibodies. Flow cytometry was conducted using an ID7000 system or BD FACS CANTO II Cytometer. The resulting data were analysed using Kaluza software (Beckman Coulter), with cell debris removed and isotype controls and single-stain controls used for gating.

### Statistical analysis

Statistical analysis was performed using GraphPad Prism software. Endpoint assays were analysed using biological replicates and data shown are mean ± standard error (SEM) unless otherwise stated. Multiple-group comparisons used one-way or two-way ANOVA with appropriate post hoc tests. Statistical significance was defined as ns, not significant, * *p* ≤ 0.05, ** *p* ≤ 0.01, *** *p* ≤ 0.001 and **** *p* ≤ 0.0001.

## Supplementary Information

Below is the link to the electronic supplementary material.


Supplementary Material 1


## Data Availability

The datasets generated and/or analysed during the current study are available from the corresponding author upon reasonable request.
