# Supplementary material for: Efficient multi-lineage cardiovascular differentiation of human pluripotent stem cells in animal serum-free conditions
Source: Sci Rep. 2026 Jun 16;16:27462. doi: 10.1038/s41598-026-56846-2 (PMC13534740; doi:10.1038/s41598-026-56846-2)
Supplement: Supplementary file 1 [file 41598_2026_56846_MOESM1_ESM.pdf]

Efficient multi-lineage cardiovascular differentiation of human pluripotent stem cells in animal serum-free conditions.

Nguyen T N Vo[1*], Kelvin Chung[1^], Aishah Nasir[1], Davor Pavlovic[2], Chris Denning[1*].

Supplemental Methods and Figures

A full description of all experimental procedures and detailed protocols is provided below; the conceptual framework and overall experimental design are summarized in the Main Methods section. For reagent and supplier details, refer to key resource table.

hIPSC Culture

The human iPSC cell lines REBL-PAT and AT1[12], used in passages 27 to 40, were maintained on Vitronectin-coated culture plastic vessels (1:100 dilution). Cells were cultured in Essential 8™ medium (E8, Life Technologies, #A1517001) and passaged every 2-3 days upon reaching 70-80% confluency using TrypLE™ Select Enzyme (1X, GibcoTM, # 12563029) for 3 minutes at 37°C. After passaging, cells were plated at a density of 20,000 - 30,000 cells per cm² in E8 medium supplemented with 10 µM Y-27632 (Tocris Bioscience #1254/10) for the initial 24 hours, followed by supplemented E8 medium. The medium was changed daily.

Monolayer cardiac differentiation of hiPSCs.

Cardiac differentiation of hiPSC lines was reported our previos study[12]. It commenced on day 0 by seeding the cells into Vitronectin-coated vessels at approximately 20-40 thousand cells/cm² in E8 medium supplemented with 10 µM Y-27632. The culture was fed with fresh E8 medium daily for the next two days. On day 2, the medium was switched to StemPro™34-Serum Free Medium (SP34, Gibco #10639011) supplemented with 2 mM L-glutamine (ThermoFisher Scientific, 25030081), 1 ng/ml BMP4 (R&D #314-BP-050). Approximately 18 hours later, the medium was replaced with SP34 containing 8 ng/ml Activin A (ActA, LifeTechnologies #PHC9564) and 10 ng/ml BMP4 (R&D, #314-BP-500/CF) for 48 hours. On day 5, the medium was switched to RPMI supplemented with B27 insulin (LifeTechnologies #A1895601) and small molecules KY0211 (R&D, #4731) and XAV939 (R&D, #3748), both at 10µM. After 48 hours, the culture medium was changed to RPMI supplemented with B27 xeno-free with insulin (termed RB27, LifeTechnologies #0080085-SA). From day 9 onwards, the culture was refreshed every 2-3 days with RB27 medium until day 15 of differentiation. hiPSC-CMs generated by the monolayer differentiation method were dissociated using TrypLE enzyme. Briefly, cells were washed twice with PBS then incubated with TrypLE for 8 – 12 minutes at 37°C in a 5% CO_2 environment. Thereafter, dissociated cardiomyocytes were collected from the flasks and washed with RPMI, followed by centrifugation at 300xg for 5 minutes and cell count.

Endothelial differentiation of hiPSCs.

Endothelial differentiation was adapted from our previous published protocol[14]. Human iPSC lines were seeded at approximately 20,000 – 40,000 cells/cm² in 10 µM Y-27632 E8 media and cultured for 24 hours before switching to RPMI 1640 supplemented with B27 minus insulin and 100 ng/ml ActA for 18 hours. The media was then changed to RPMI 1640 supplemented with B27 minus insulin and 5 ng/ml BMP4 + 1 µM CHIR99021 (Selleckchem, S2924) for 24 hours. Cells were subsequently treated with SP34 medium supplemented with 2 mM L-glutamine, 50 µg/ml ascorbic acid (Sigma, A8960-5G), 10 ng/ml BMP4, 5 ng/ml FGF2 (R&D, 3718-FB-100), and 300 ng/ml VEGF (QKine, QK048-0100) for 72 hours at 37°C, 5% CO_2. On day 7, cells were dissociated, washed with PBS, and treated with 1 mL TryPLE enzyme for 7 minutes at 37°C, 5% CO_2. Cells were removed from the flask, diluted with 4 mL pre-warmed SP34 serum-free medium, and centrifuged at 200 g for 3 minutes. The cell count was performed, and cells were replated at 10,000 cells per cm² in Endothelial Growth Medium (Lonza, # CC-3124) supplemented with Panexin NTA (PAN Biotech, #P04-95750P), but not Fetal Bovine Serum FBS

and Bovine Brain Extract (BBE), 20 ng/ml VEGF + 20 ng/ml FGF2. From day 7 onward, cells were cultured in animal serum-free EGM medium supplemented with 20 ng/ml VEGF + 20 ng/ml FGF2. The medium was refreshed every 2 days.

Cardiac fibroblast differentiation of hiPSCs.

The method for differentiating quiescent hiPSC-CFs adapted from Zhang et al., 2019[13]. Initially, all hIPSC cells were seeded on a Vitronectin-coated six-well plate at densities ranging from 20,000 to 30,000 cells/cm² and incubated in E8 medium supplemented with 10 μM Y-27632 at 37°C. After 48 hours, the medium was switched to RPMI 1640 supplemented with B27 minus insulin and 6 μM CHIR99021 for another 48 hours, followed by 24 hours in RPMI 1640 with B27 minus insulin. Subsequently, the cells were treated with RPMI 1640 containing B27 minus insulin and 5 μM IWR1 for 48 hours. Cells were then dissociated using TryPLE enzyme for 3 minutes at 37°C, 5% CO_2, then diluted with RPMI 1640, and centrifuged at 200xg for 3 minutes. The resuspended cells were cultured at 12,000 cells/ cm^2 in Advanced DMEM/F-12 with 5 μM CHIR99021 and 2 μM Retinoic Acid in a Vitronectin-coated T25 flask for three days, followed by a change to Advanced DMEM/F-12 for four additional days. Cells were then passaged and cultured in Fibroblast Growth Medium 3 supplemented with Panexin NTA but not FBS, 10 ng/mL FGF2 and 10 μM TGF-β receptor inhibitor SB431542, changing the medium every 48 hours for six days. Post-cultivation, cells were assessed for purity using immunocytochemistry and prepared for long-term storage by cryopreservation in serum-free media after reaching 70-80% confluency.

Myofibroblast activation assay

To induce myofibroblast activation, hiPSC-derived cardiac fibroblasts at day 20 post-differentiation were dissociated using TryPLE enzyme then plated into 96 well plate at 12,000 cells/ cm^2. The medium was refreshed next day. On day 22, the cells treated with recombinant human TGFβ1 (10 ng/mL) for 48 hours. Where indicated, cells were co-treated with the TGFβ receptor inhibitor SB431542 (10 μM) to suppress pathway activation. Vehicle-treated cells was also supplemented with SB431542 and served as controls. Following treatment, cells were processed for immunostaining and quantitative analysis of αSMA expression.

Generate EHTs

Engineered heart tissues (EHTs) were generated following protocols[12,19]. Initially, a 2% ultra-pure agarose solution (Invitrogen #15510-027) was solidified around polytetrafluoroethylene (PTFE) spacers (EHT Technologies #C0002) in the wells of a 24-well cell-culture plate (Thermo Fisher Scientific #142475) for 15 minutes to create casting molds. These spacers were then replaced with polydimethylsiloxane (PDMS) racks (EHT Technologies #C0001) to extend PDMS posts into each mold.

A master mix was prepared containing $1x10^6$ CMs, CFs, and ECs in a 70:15:15 ratio, supplemented with 2XDMEM (ThermoFisher, #52100-021), 10 μM Y-27632, 5 mg/mL fibrinogen (Scientific Laboratory Supplies #F4883-500MG), and NKM medium composed of DMEM (PAN-Biotech #P04-01500), 1% penicillin/streptomycin (P/S, Thermo Fisher Scientific #15070063), 2 mM L-glutamine, with options of 10% fetal calf serum (Sigma #N4762), human serum (Sigma #H4522), or Panexin NTA. This mixture was combined with 100U/mL thrombin (Sigma #605195) and pipetted into the apertures between the PDMS posts, followed by a 2-hour incubation at 37°C, 5% CO_2. Subsequently, NKM medium was added to cover the wells, with further incubation for 20-30 minutes under the same conditions. The EHTs were transferred to new 24-well plates containing EHT medium enriched with DMEM, 10% horse serum (ThermoFisher, #26050088), human serum, or Panexin NTA, along with 10 μg/mL insulin (Sigma #I9278), 33 μg/mL aprotinin (Sigma, #A1153), and 1% P/S. From day 3 post-casting, EHTs were nourished every other day with EHT medium containing varying concentrations of horse serum, human serum, or Panexin NTA for 3-4 weeks.

Immunocytochemistry

Cells were washed once with PBS and then fixed in 4% Paraformaldehyde (PFA, Sigma) at room temperature (RT) for 12 minutes. After fixation, cells were washed again with PBS before permeabilization with 0.05% Triton X-100 (Sigma) in PBS for 15 minutes at RT. Subsequent to permeabilization, cells were rinsed with 0.5% Tween 20 in PBS and incubated with 4% goat serum (Sigma) in PBS (as a blocking solution) for one hour at RT to prevent nonspecific antibody binding. Overnight incubation with primary antibodies was then performed at 4°C in the blocking solution. The following day, cells were washed three times with 0.5% Tween-20 in PBS, each wash lasting 5 minutes, and then incubated with Alexa Fluor secondary antibodies (Life Technologies) in blocking solution for one hour at RT. Cells were subsequently washed three times with 0.5% Tween-20 in PBS for 5 minutes per wash. Finally, nuclei were stained with 0.5 μg/ml DAPI (Sigma #D9542) for 20 minutes and then rinsed with 0.5% Tween 20 in PBS. For quantification and measurement of immunocytochemistry results, images conducted from Operetta® High Content Imaging System (PerkinElmer), were processed and analysed using Harmony® High Content Imaging and Analysis Software (PerkinElmer).

Flow Cytometry

Cells were collected using TryPLE as per the referenced protocol at 200xg for 3 minutes in a cold centrifuge maintained at 4°C. The cell pellets were washed with ice-cold PBS, and then fixed in 4% paraformaldehyde, permeabilised with 0.05% Triton X-100, blocked with 1% BSA in PBS (termed PBA). The cell pellets were then transferred to flow cytometry tubes or V-bottom 96-well plates. The pellets were thoroughly mixed with the antibodies prepared in cold PBA, following the dilutions specified in the accompanying table. The pellets were incubated with conjugated antibodies 30 minutes in the dark at 4°C. While the incubation with primary antibodies can be done overnight at 4°C following with 30 minutes incubation of secondary antibodies. The samples were then centrifuged at 300xg for 5 minutes, and the supernatant was discarded. This step was repeated after resuspending the cell pellets in cold PBA to ensure complete removal of residual antibodies. Flow cytometry was conducted using an ID7000 system or BD FACS CANTO II Cytometer, and the resulting data were analyzed using Kaluza software (Beckman Coulter), with cell debris removed and isotype controls and single-stain controls used for gating.

Statistical analysis

Statistical analysis was performed using GraphPad Prism software. Endpoint assays were analysed using biological replicates and data shown are mean ± standard error (SEM) unless otherwise stated. Multiple-group comparisons used one-way or two-way ANOVA with appropriate post hoc tests. Statistical significance was defined as ns, not significant, * $p \leq 0.05$, ** $p \leq 0.01$, *** $p \leq 0.001$ and **** $p \leq 0.0001$.

Supplemental Figures

Supplement Figure S1. Cardiac fibroblast differentiation optimisation using the REBL-PAT hiPSC line. (A) Schematic of the modified cardiac fibroblast differentiation protocol. (B) Representative cell morphology during REBL-PAT-derived cardiac fibroblast differentiation under serum-based and serum-free conditions. Scale bar: 500 μm. (C) Representative immunostaining showing myofibroblast activation in REBL-PAT-derived cardiac fibroblasts differentiated using serum-based medium or serum-free medium with Panexin NTA. Nuclei (DAPI in blue), COL-I(green), α-SMA (red), Scale bar = 100 μm. (D) Quantification of cardiac fibroblast marker-positive cells under different seeding-density conditions during the modified serum-free protocol with Panexin NTA. This optimisation was performed using COL-I and DDR2. (E) Quantification of αSMA-positive cells as an indicator of myofibroblast activation. Data are for TGFβ1 alone, SB431542 alone or combined TGFβ1 and SB431542; the SB431542 alone condition was used as a baseline of activation. Data are presented as mean ± SEM, N = 3 biological replicates. Two-way ANOVA with Tukey's multiple-comparisons test. Created in BioRender. Vo, N. (2026) https://BioRender.com/ma2votl.

Supplement Figure S2. Endothelial differentiation optimisation and endothelial network-like organisation. (A) Schematic of the modified endothelial differentiation protocol. (B) Representative cell morphology during endothelial differentiation under serum-based and serum-free conditions using Panexin NTA. Scale bar: 500 μm. (C) Representative flow cytometry plots for endothelial markers CD31, CD144/VE-cadherin and CD34 on day 14 of differentiation. (D) Quantification of endothelial differentiation efficiency under different seeding-density conditions in the serum-free Panexin protocol. Data are presented as mean ± SEM, N = 3 biological replicates. Two-way ANOVA with Tukey's multiple-comparisons test. (E) Representative co-culture images showing endothelial network-like organisation generated from REBL-PAT- and AT1-derived hiPSC-ECs and hiPSC-CFs. CD31-positive endothelial cells are shown in green, vimentin-positive stromal cells in yellow, and nuclei are shown by DAPI in blue. Scale bars 200 μm. Created in BioRender. Vo, N. (2026) https://BioRender.com/ma2votl.

Supplementry figure S3. Evaluation of serum replacement strategies in CM-only EHTs. (A) Schematic and timeline for cardiomyocyte differentiation and EHT experimentation. (B) Schematic showing the generation of EHTs using Panexin NTA and human serum to replace the FCS component in the EHT media. (C) Contraction analysis using the EHT platform and Consulting Team Machine Vision (CTMV) software, with a diagram explaining the main parameters for contractility analysis, including contraction force, contraction time, and relaxation time. (D) Observations of poor attachment and low yield of EHTs under serum-free conditions during tissue fabrication and maintenance day 14. Created in BioRender. Vo, N. (2026) https://BioRender.com/ma2votl.

Supplement Figure S4. Quality control of cell populations used for triculture EHT generation. Quality control results indicate high purity (>85%) for each cell type, including cardiomyocytes (CMs), cardiac fibroblasts (CFs), and endothelial cells (ECs) using the AT1 hiPSC line. Created in BioRender. Vo, N. (2026) https://BioRender.com/ma2votl.

Supplementary Figure S5. Limited recovery of contractile function in triculture EHTs cultured with Panexin following removal of SB431542. Contraction traces of triculture EHTs cultured in Panexin-based medium in the absence of the TGFβ receptor inhibitor SB431542. Despite removal of SB431542 and the presence of cardiac stromal cells, triculture EHTs cultured with Panexin showed only modest contractile forces (~0.05 mN), which remained substantially lower than those observed in human serum conditions. Data are shown from two independent EHT fabrications (N = 2 biological replicates), each containing 2–4 technical replicates, and represent preliminary validation experiments. Created in BioRender. Vo, N. (2026) https://BioRender.com/ma2votl.

Supplementary Figure S6. Comparison of contractile performance of triculture EHTs cultured with and without SB431542. Comparison of contraction force (mN) of triculture

EHTs under different serum conditions (10% horse serum, 10% human serum, and 1% human serum) in the presence or absence of the TGFβ receptor inhibitor SB431542. Contractile measurements were recorded at day 14, day 21, and day 28 post-EHT formation. data are presented as mean ± SEM. Datapoints are technical EHT replicates to show experimental variation, but statistical comparisons were performed using biological replicates (N = 3). Two-way ANOVA with Sidak's multiple-comparisons test; colour-coded according to serum condition, compared contraction force with and without SB431542 at each time point; ns, not significant; *$p < 0.05$, **$p < 0.01$. Created in BioRender. Vo, N. (2026) https://BioRender.com/ma2votl.

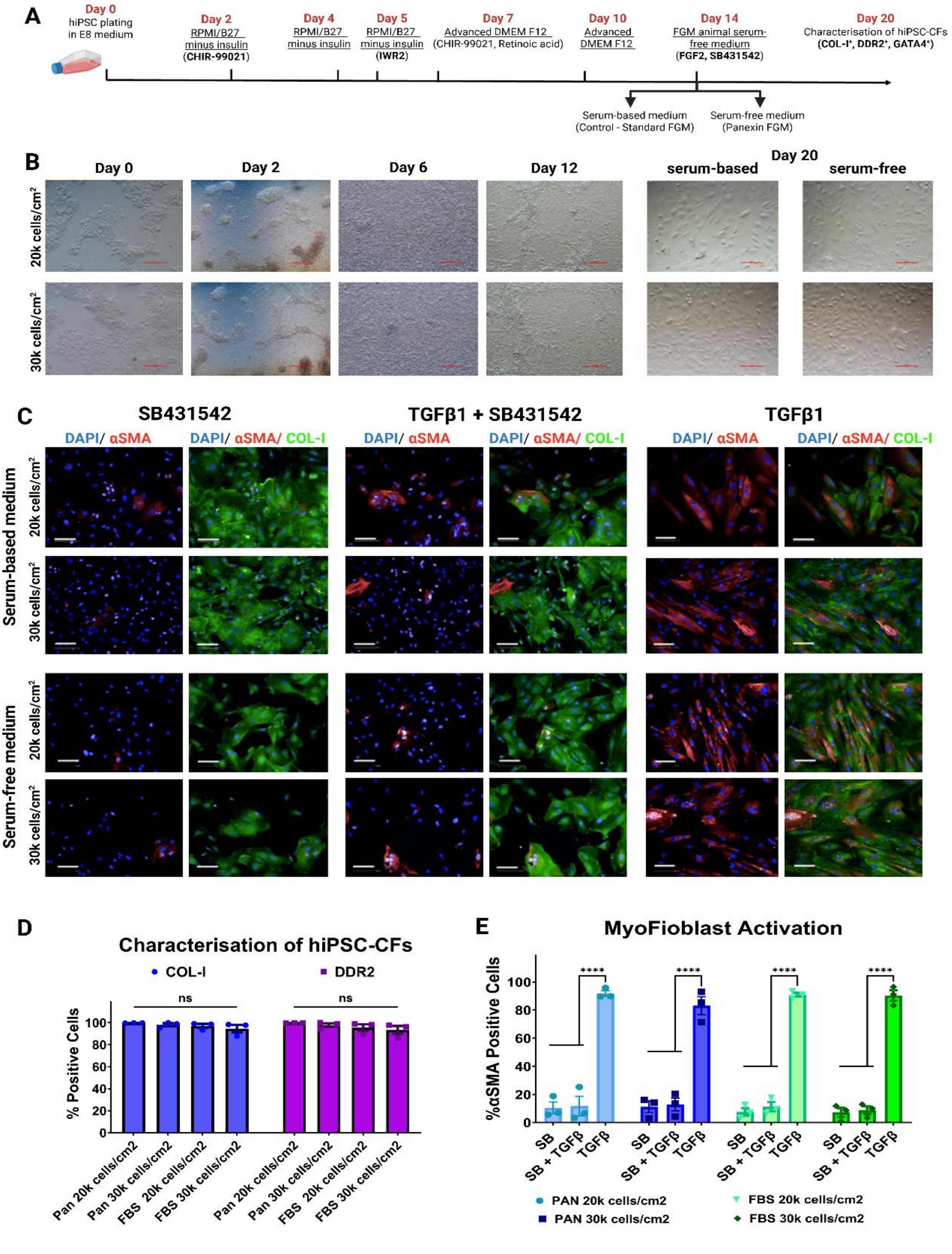

Supplement Figure S1.

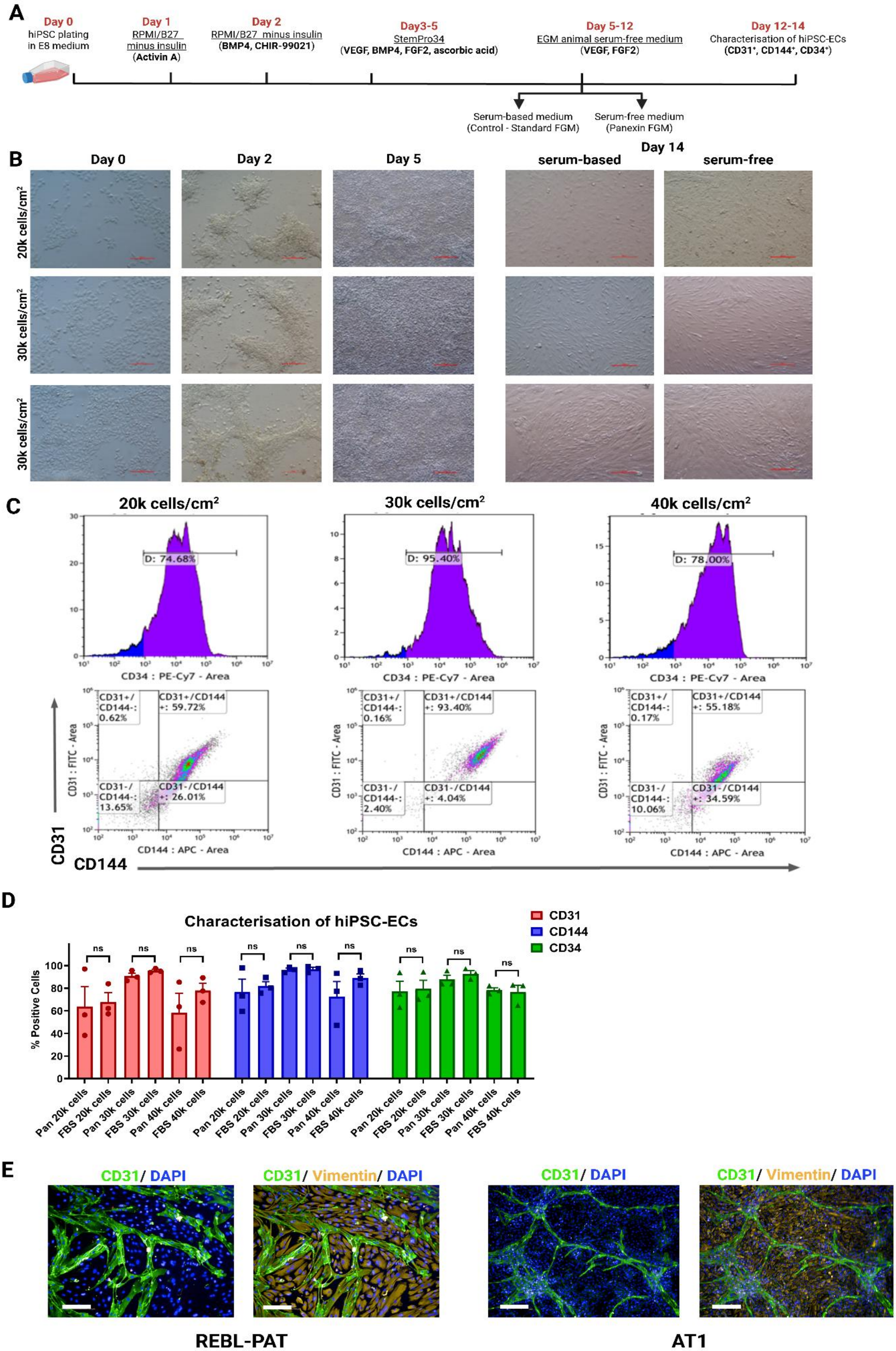

Supplement Figure S2.

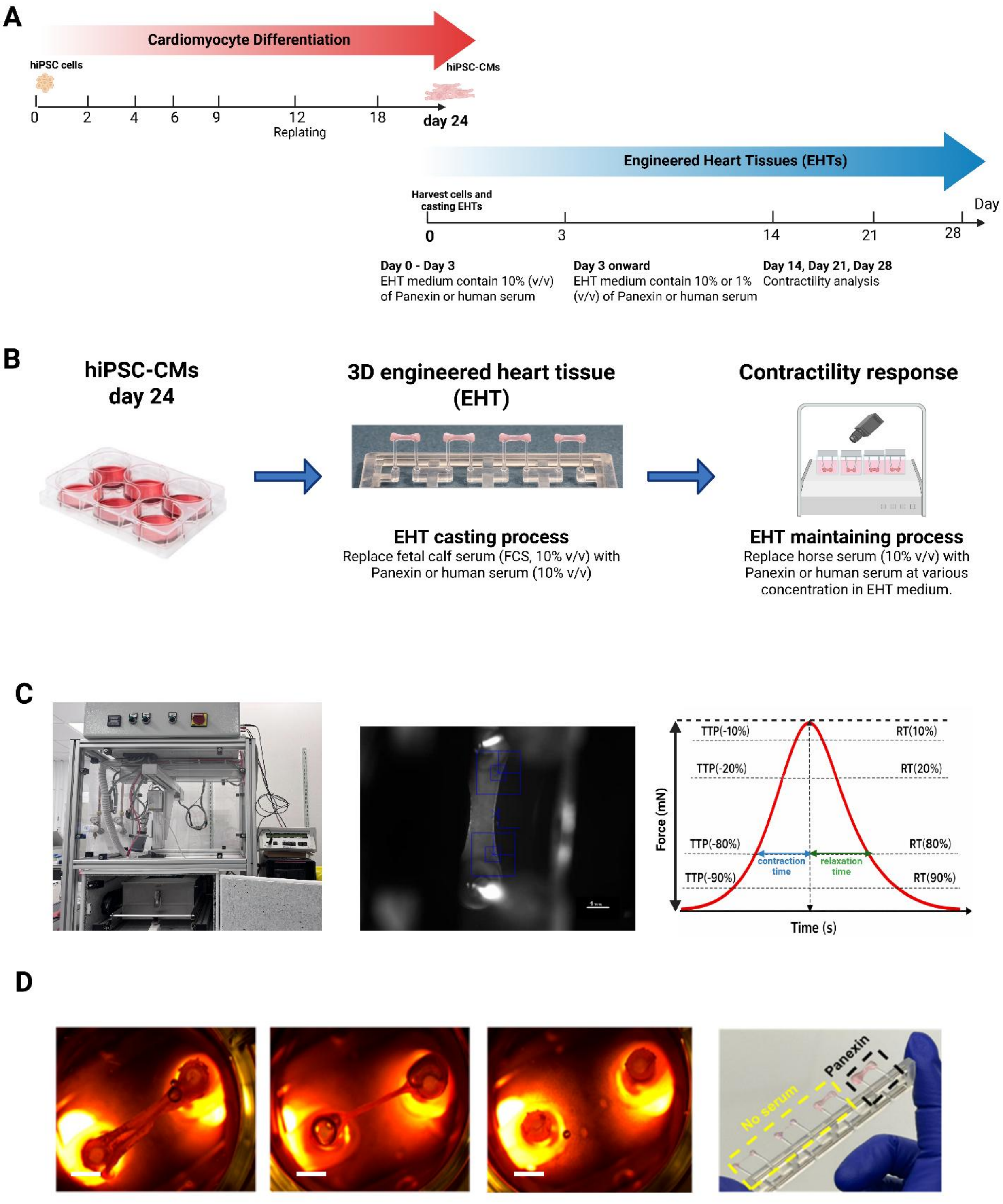

Supplement Figure S3

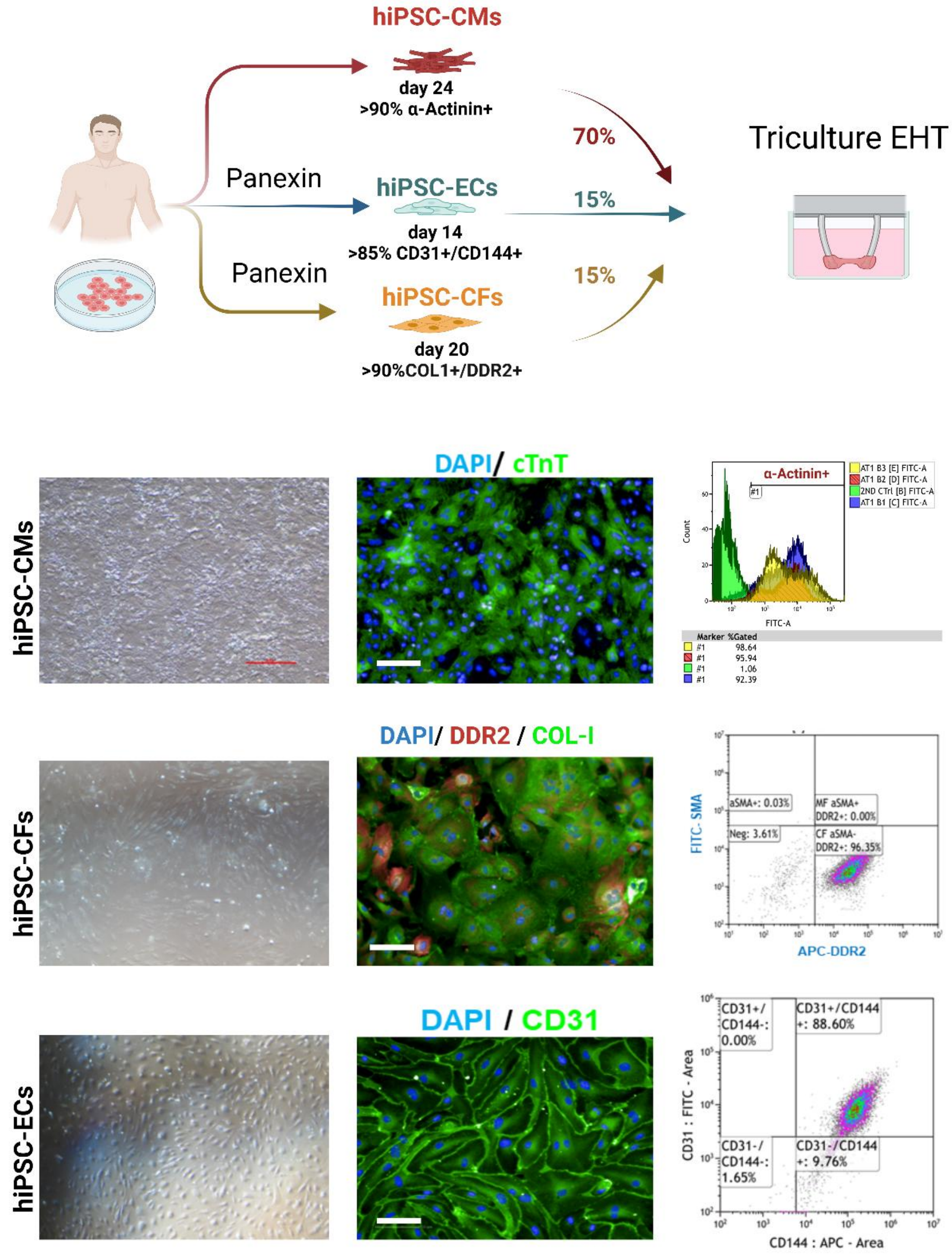

Supplement Figure S4

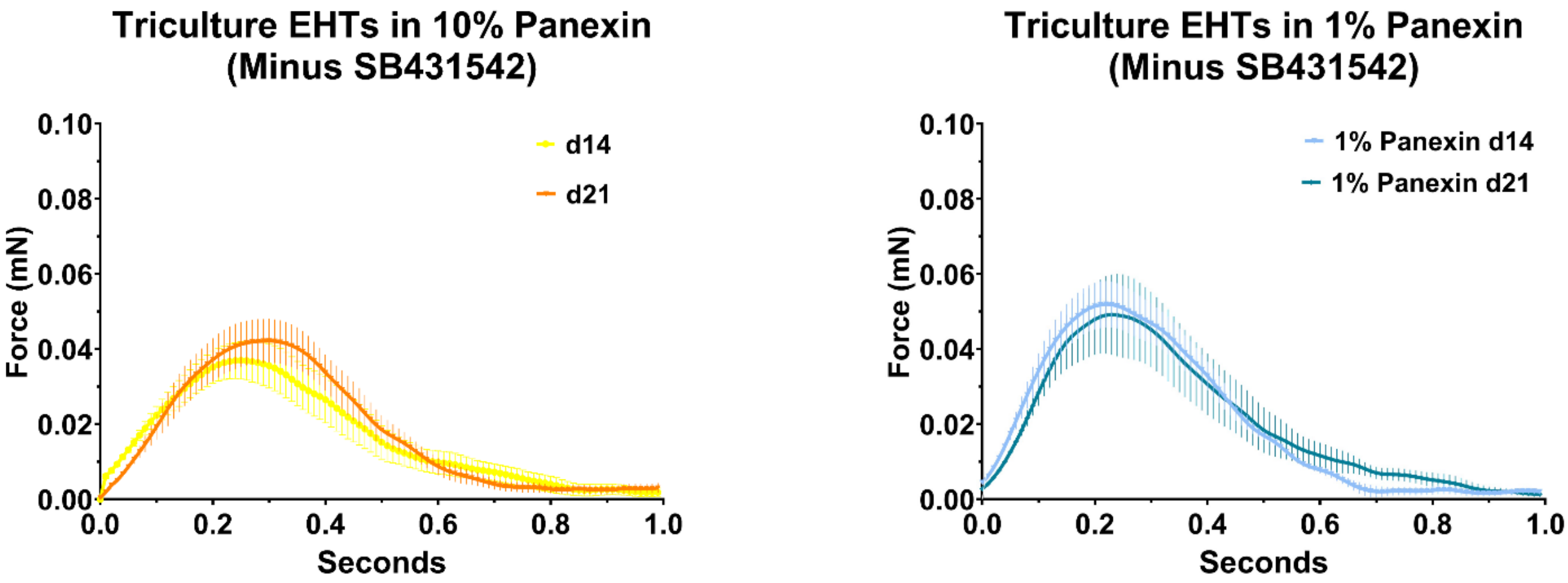

Supplement Figure S5

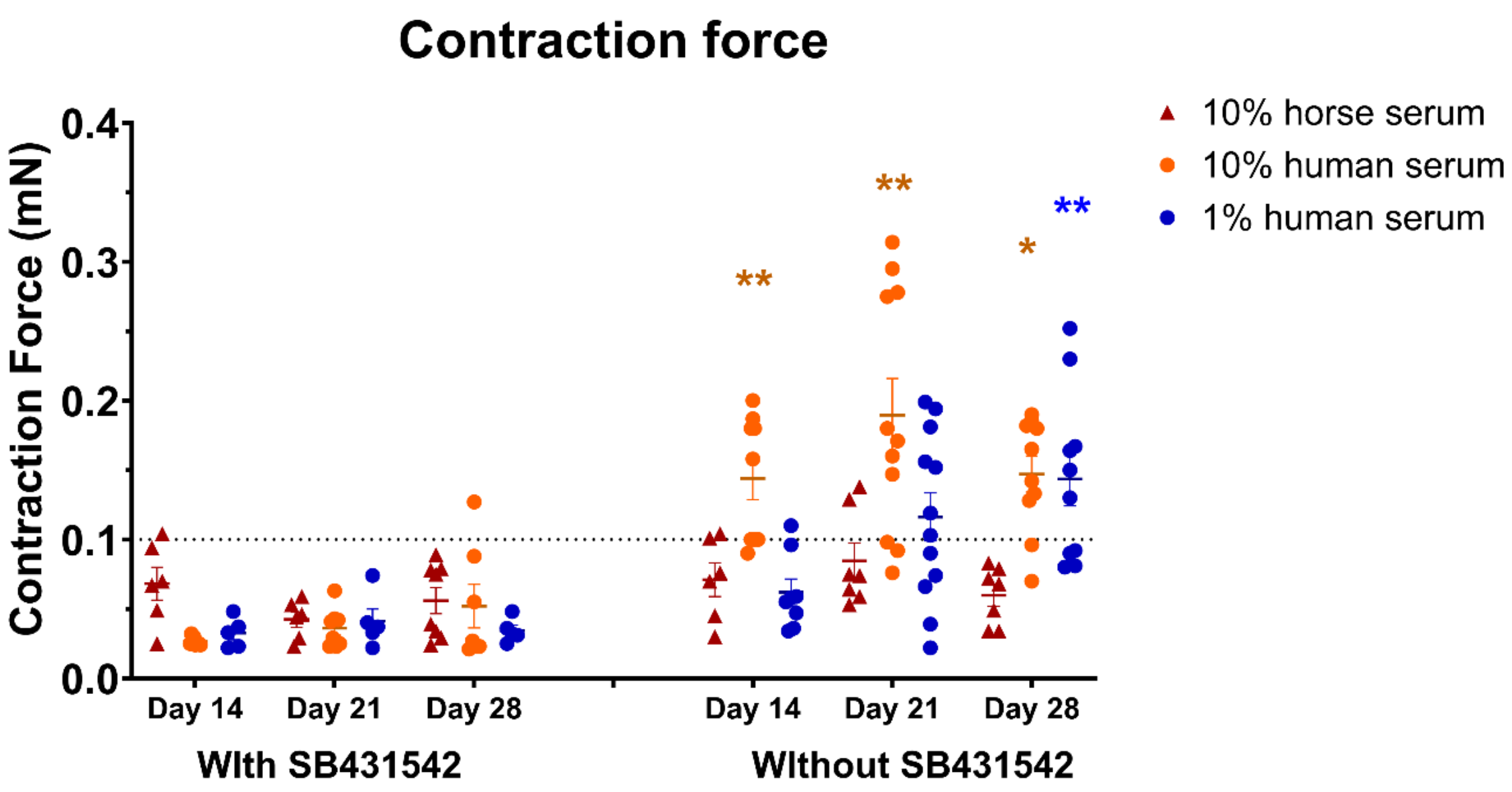

Supplement Figure S6

Supplementary Table S1. Key resource table. List of reagents, media, and supplements used in this study

Reagent	Company and Catalogue Number
Advanced DMEM/F-12	ThermoFisher Scientific, 12634010
Alexa Flour 488 Goat anti Mouse	Thermo Fisher Scientific, A11029, working dilution 1:1000
Alexa Flour 488 Goat anti Rabbit	Thermo Fisher Scientific, A11008, working dilution 1:1000
Alexa Flour 488 Goat anti RabbitCD34 Monoclonal Antibody (4H11), PE-Cyanine7, eBioscience™	Thermo Fisher Scientific, 25-0349-42, working dilution 1:50
Alexa Flour 647 Goat anti Mouse	Thermo Fisher Scientific, A21235, working dilution 1:1000
Anti-alpha smooth muscle Actin antibody	Abcam, ab7817, working dilution 1:1000
Anti-Cardiac Troponin T antibody	Abcam, ab45932, working dilution 1:400
Anti-Collagen I antibody	Abcam, ab34710, working dilution 1:1000
Anti-DDR2 antibody	Abcam, ab63337, working dilution 1:1000
Anti-GATA-4 Antibody (G-4)	Santa Cruz, sc-25310, working dilution 1:1000
Aprotinin from bovine lung	Sigma, A1153
Ascorbic acid	Sigma, A8960-5G
B-27™ Supplement (50X), minus insulin	ThermoFisher Scientific, A1895601
B-27™ Supplement (50X), serum free	ThermoFisher Scientific, 17504044
BD Pharmingen™ FITC Mouse Anti-Human CD31	BD Biosciences, 555445, working dilution 1:20
Bovine Albumin Fraction V (7.5% solution)	Thermo Fisher Scientific, 15260037
Ca^{2+} / Mg^{2+} free Phosphate Buffer Saline	PBS, Gibco, 14190-094
CD144 (VE-cadherin) Monoclonal Antibody (16B1), APC, eBioscience™	Thermo Fisher Scientific, 17-1449-42, working dilution 1:50
CHIR99021	Selleckchem, S2924
Corning® Matrigel® Basement Membrane Matrix, LDEV-free	CORNING, 354234
CryoStor® CS10	STEMcell Technology, 07959
Di-amino phenyl-indole	Sigma, D9542
Dimethyl sulfoxide	Sigma, D2650
DMEM, powder, high glucose	Thermo Fisher Scientific, 52100-021
Donkey anti-Sheep IgG (H+L) Cross-Adsorbed Secondary Antibody AF 488	Thermo Fisher Scientific, A11015, working dilution 1:1000
Essential 8™ medium	Thermo Fisher Scientific, A1517001
Fibrinogen from human plasma	Scientific Laboratory Supplies, F4883-500MG
Fibroblast Growth Medium 3	(PromoCell, C-23025)
Horse Serum, heat inactivated	Thermo Fisher Scientific, 26050088
Human Activin A Recombinant Protein	ThermoFisher Scientific, PHC9564
Human CD31/PECAM-1 Antibody	R&D Systems, AF806, working dilution 1:1000
Human serum	Sigma, H4522
Human TGF-beta 1 Recombinant Protein	PeproTech, 100-21-10UG
Human VEGF-165 Recombinant Protein	QKine, QK048-0100
Insulin Solution, human recombinant	Sigma, I9278
IWR-1	Sigma-Aldrich, I0161
KY 02111	TOCRIS, 4731
L-Glutamine (200 mM)	ThermoFisher Scientific, 25030081
Lonza EGM™ Endothelial Cell Growth Medium BulletKit	Lonza, CC-3121
Monoclonal Anti-A-Actinin	Sigma, A7811, working dilution 1:800
Mouse IgG1, κ Isotype Control	BD Biosciences, 555748
Mouse IGG1K isotype control APC	Thermo Fisher Scientific, 17-4714-82
Mouse IGG1K isotype control PECY-7	Thermo Fisher Scientific, 25-4714-80
Newborn Calf Serum	Sigma, N4762
Pan DMEM	PanTechnology, P04-01500
Panexin NTA Pharma Grade	PANBiotech, P04-95750P
Penicillin-Streptomycin	Thermo Fisher Scientific, 15070063
Polydimethylsiloxane racks	EHT Technologies C0001
Polytetrafluorethylene spacers	EHT Technologies, C0002

Recombinant Anti-Vimentin antibody	Abcam, ab92547, working dilution 1:1000
Recombinant Human BMP-4 Protein	R&D Systems, 314-BP-010/CF
Recombinant Human FGF basic/FGF2/bFGF (146 aa) Protein	R&D, 3718-FB-100
Retinoic Acid	Sigma-Aldrich, R2625-50MG
RPMI 1640 Medium	ThermoFisher Scientific, 11875093
SB 431542	Selleckchem, S1067
StemPro™-34 SFM (1X)	ThermoFisher Scientific, 10639011
Thrombin, Human Plasma	Sigma, 605195
TrypLE™ Select (1X)	Thermo Fisher Scientific, 12563029
UltraPure™ Agarose	Invitrogen, 15510-027
Vitronectin-N	VN, Lifetech #A14700
XAV 939	TOCRIS, 3748
Y-27632 (Dihydrochloride)	Tocris Bioscience #1254/10
